# Low antibodies against *Plasmodium falciparum* and imbalanced pro-inflammatory cytokines are associated with severe malaria in Mozambican children: a case–control study

**DOI:** 10.1186/1475-2875-11-181

**Published:** 2012-05-30

**Authors:** Eduard Rovira-Vallbona, Gemma Moncunill, Quique Bassat, Ruth Aguilar, Sonia Machevo, Laura Puyol, Llorenç Quintó, Clara Menéndez, Chetan E Chitnis, Pedro L Alonso, Carlota Dobaño, Alfredo Mayor

**Affiliations:** 1 Barcelona Centre for International Health Research, (CRESIB, Hospital Clínic-Universitat de Barcelona), Barcelona, Spain; 2 Centro de Investigação em Saúde de Manhiça (CISM), Maputo, Mozambique; 3 International Centre for Genetic Engineering and Biotechnology (ICGEB), New Delhi, India

**Keywords:** *Plasmodium falciparum*, Children, Severe malaria, Antibodies, Cytokines

## Abstract

**Background:**

The factors involved in the progression from *Plasmodium falciparum* infection to severe malaria (SM) are still incompletely understood. Altered antibody and cellular immunity against *P. falciparum* might contribute to increase the risk of developing SM.

**Methods:**

To identify immune responses associated with SM, a sex- and age-matched case–control study was carried out in 134 Mozambican children with SM (cerebral malaria, severe anaemia, acidosis and/or respiratory distress, prostration, hypoglycaemia, multiple seizures) or uncomplicated malaria (UM). IgG and IgM against *P. falciparum* lysate, merozoite antigens (MSP-1_19_, AMA-1 and EBA-175), a Duffy binding like (DBL)-α rosetting domain and antigens on the surface of infected erythrocytes were measured by ELISA or flow cytometry. Plasma concentrations of IL-12p70, IL-2, IFN-γ, IL-4, IL-5, IL-10, IL-8, IL-6, IL-1β, TNF, TNF-β and TGF-β1 were measured using fluorescent bead immunoassays. Data was analysed using McNemar’s and Signtest.

**Results:**

Compared to UM, matched children with SM had reduced levels of IgG against DBLα (*P* < 0.001), IgM against MSP-1_19_ (*P* = 0.050) and AMA-1 (*P* = 0.047), TGF-β1 (*P* <0.001) and IL-12 (*P* = 0.039). In addition, levels of IgG against *P. falciparum* lysate and IL-6 concentrations were increased (*P* = 0.004 and *P* = 0.047, respectively). Anti-DBLα IgG was the only antibody response associated to reduced parasite densities in a multivariate regression model (*P* = 0.026)*.*

**Conclusions:**

The lower levels of antibodies found in children with SM compared to children with UM were not attributable to lower exposure to *P. falciparum* in the SM group. IgM against *P. falciparum* and specific IgG against a rosetting *Pf*EMP1 domain may play a role in the control of SM, whereas an imbalanced pro-inflammatory cytokine response may exacerbate the severity of infection. A high overlap in symptoms together with a limited sample size of different SM clinical groups reduced the power to identify immunological correlates for particular forms of SM.

## Background

In countries endemic for *Plasmodium falciparum*, severe malaria (SM) was responsible for approximately 0.7 million deaths in 2010, predominantly children under five years of age [1]. Reduction of case fatality rates and morbidity is hampered by an incomplete understanding of the parasite and host factors involved in the pathophysiology of life-threatening disease. Progression from infection to severe clinical malaria is probably multi-factorial, including sequestration of infected erythrocytes (IEs) in vital organs and mechanical obstruction of blood flow, limited malaria-specific antibody immunity and deregulated inflammatory responses to *P. falciparum*[2].

Antibodies targeting blood stage parasite antigens are acquired after exposure to *P. falciparum*[3] and may contribute to protection from SM by reducing parasite density [4]. Specifically, antibodies against *P. falciparum* erythrocyte membrane protein 1 (*Pf*EMP1) have been suggested to block formation of rosettes [5] and cytoadhesion of IEs to specific host receptors [6]. Several studies have reported associations between antibody responses and risk of SM in young children [7-12]. However, conclusions are contradictory, some showing low immunoglobulin G (IgG) levels in children with severe anaemia (SA) [7,10] compared to uncomplicated malaria (UM), others reporting no associations [9] or even opposite trends for cerebral malaria (CM) [7]. In addition, the role of immunoglobulin M (IgM) antibodies in children with SM has been much less explored [9,12], although reduced IgM responses have been observed in adults with CM [13,14].

In addition to antibodies, cytokines such as interleukin (IL)-12, interferon (IFN)-γ and tumor necrosis factor (TNF) have been shown to be involved in the control of *P. falciparum* infection [15-17]. However, the excessive production of pro-inflammatory cytokines, such as TNF and IL-6, may damage host tissues, increase the expression of adhesion molecules on endothelial cells and enhance parasite cytoadhesion [18]. The regulation of pro-inflammatory cytokines production by IL-10 or transforming growth factor (TGF)-β1 seems to be a key factor in preventing acute pathology [19-21] and, overall, the fine balance between pro-inflammatory versus immuno-regulatory cytokines is suggested to determine the outcome of *P. falciparum* infection [22].

Combined information on both antibody and cytokine responses is needed to understand the role of immunity in the progression of malaria infection to SM, and to develop rational strategies that reduce mortality and morbidity associated to SM. Remarkably, there is scarce data on antibody responses in children with signs of severity other than SA and CM such as prostration, acidosis and/or respiratory distress (ARD) or multiple seizures (MS), which are among the most prevalent forms of SM in some endemic areas [23]. In the present study, the association of SM with low antibody responses and an exacerbated pro-inflammatory cytokine response was tested in Mozambican children. IgG and IgM against parasite lysate, merozoite antigens and *P. falciparum* antigens on the surface of IEs, as well as plasma cytokines and chemokines, were compared between children with different clinical presentations of SM and children with UM, matched by sex and age.

## Methods

### Study area

The area of study was located in the Manhiça District, southern Mozambique. Detailed descriptions of the area have been reported elsewhere [24]. Briefly, Manhiça is characterized by a perennial malaria transmission with some seasonality and of moderate intensity, mostly attributable to *P. falciparum*. The majority of SM cases occur in children under five years of age. Prostration, ARD and SA are the most common clinical presentations, with coma being infrequent and slightly shifted to older children [23].

### Study design and patients

Between April and November 2006, a sex and age (+/−3 months) matched case–control study was conducted, with a primary aim of characterizing the cytoadhesion phenotypes of *P. falciparum* isolates [25]. Children under five years of age attending the Manhiça District Hospital with a clinical diagnosis of *P. falciparum* malaria were recruited after written informed consent was given by their parents or guardians. Clinical malaria was defined as the presence of fever (axillary temperature ≥37.5 °C) with an asexual *P. falciparum* parasitaemia of ≥500/μL by microscopic examination of Giemsa-stained blood smears; this definition of malaria has a sensitivity and specificity of >90% in children from Manhiça [26]. Cases were children presenting with clinical malaria and at least one of the following definitions of SM [27]: CM (Blantyre Coma Score ≤2), SA (packed cell volume <15% or hemoglobin <5 g/dL), ARD (lactate >5 mM and/or chest indrawing or deep breathing), prostration (inability to sit or breastfeed in children old enough to do so), hypoglycaemia (blood glucose <2.2 mM) and MS (≥2 convulsions in the preceding 24 h) . Controls were outpatient children with malaria not showing any of the mentioned signs of severity and able to take oral medication (UM group). All patients were reviewed by the study pediatrician to confirm that malaria was the sole or principal cause of the disease. Children with positive bacteraemia were excluded from the study. Malnutrition was defined as the presence of marasmus or kwashiorkor by clinical examination or as a mid-upper arm circumference <12.5 cm in children >12 months of age. SM patients were admitted and treated with intravenous quinine until able to switch to oral therapy, while UM controls were treated following Mozambican national guidelines at that time (artesunate plus sulphadoxine-pyrimethamine). The study was approved by the National Mozambican and the Hospital Clinic of Barcelona Ethics Review Committees.

### Sample collection

Before treatment, peripheral blood was taken by venipuncture into a tube containing lithium heparin, and 2 drops of blood were spotted onto filter paper (Schleicher & Schuell; n° 903^TM^). Biochemical determinations (alanine aminotransferase, bilirubin and creatinine) and full blood counts were performed using Vitros DT60 and Sysmex Kx21 analyzers, respectively. Lactate was determined using Lactate Pro® (Fact Canada) at the bed side. After centrifugation, plasma was stored at −80 °C, erythrocyte pellets were Buffy-coat depleted, washed 3 times in phosphate-buffered saline (PBS) and cryopreserved in glycerolyte solution.

### Parasite density and multiplicity of infection

DNA was extracted from a 50 ml blood drop onto filter paper with QIAamp DNA Mini Kit (Qiagen), and resuspended in 150 ml of water. Five ml of DNA samples, tested in triplicate, were used to measure parasite density by a real-time quantitative PCR (qPCR) targeting the *P. falciparum* 18 S ribosomal RNA gene, as described elsewhere [28]. Multiplicity of infection (MOI) was determined by nested PCR-typing of the polymorphic regions of *msp-1* and *msp-2* genes [29], and estimated as the highest number of *msp-1* or *msp-2* alleles in the sample.

### Enzyme-linked immunosorbent assays (ELISA)

IgG and IgM antibodies were measured against the recombinant proteins merozoite surface protein 1 (MSP-1_19_, 19 kD fragment, 3D7 [30]), erythrocyte binding antigen 175 (EBA-175, F2 region, CAMP [31]), apical membrane antigen 1 (AMA-1, full ectodomain, 3D7 [32]) and a Duffy binding like alpha (DBLα) domain from a *Pf*EMP1 involved in rosetting through adhesion to complement receptor 1 (*R29var1* minimal domain, [33]), all produced at the International Centre for Genetic Engineering and Biotechnology (New Delhi, India). High-binding 96-well microplates (Nunc Maxisorp) were coated with 200 ng/well of recombinant protein in 0.05 M carbonate-bicarbonate buffer. Plates were washed with 0.05% Tween-20 in PBS (PBS-Tween), blocked with 2% bovine serum albumin (BSA) in PBS-Tween for 8 h at 4 °C and washed again. One hundred μl of plasma at 1/200 dilution along with positive (pool from 8 hyper-immune Mozambican adults) and negative controls (9 unexposed Europeans) were added in duplicate to wells. After incubation overnight at 4 °C, peroxidase-conjugated goat anti-human IgG or IgM secondary antibodies (Sigma) were added at 1/30000 and 1/2000, respectively. After 1 h of incubation and washing, 100 μl/well of a phosphate solution with 0.012% of H_2_O_2_ substrate and *o*-phenylendiamine chromagen were added for 5 min and the colorimetric reaction was stopped with 25 μl/well of H_2_SO_4_. Specific reactivity of plasmas was obtained as optical density (OD) values measured at 492 nm (Multiskan EX, Labsystems) and normalized by dividing OD of each sample by the OD of the positive control run in each plate.

To measure antibodies to *P. falciparum* lysate, the 3D7 and HB3 clones (provided by the Malaria Research and Reference Reagent Resource Center, MR4) from asynchronous cultures at 5% parasitaemia were homogenized in PBS at 1% haematocrit by a minimum of three freeze-thawing steps and mixed (1:1). A lysate of uninfected erythrocytes was used to determine unspecific recognition. Coating was done with 50 μl of erythrocyte extract and plasma was added at 1/1600 dilution. Reaction was performed as described above. OD of uninfected lysate wells was subtracted from OD of infected lysate wells, and values were normalized against the positive control run in each plate.

### Antibodies to IEs surface antigens

Five paediatric isolates (three from SM patients [MOZ1, MOZ2 and MOZ4] and two from UM patients [MOZ3 and MOZ5]) and two placental isolates from O blood group individuals [25,34], were tested for antibody recognition. MOZ2 was adapted to *in vitro* culture, whereas the remaining isolates were used in antibody measurements without *in vitro* expansion. IgG were also measured against four laboratory clones with different receptor binding phenotypes: R29 (rosetting, [35]), ITG_ICAM_ (ICAM-1, [36]), FCR3_CSA_ (CSA, [37]) and E8B_CD36_ (CD36, [38]). The study samples, plus negative and positive controls, were tested blindly in a single assay against each parasite. Briefly, cryopreserved ring-stage parasites were thawed in a sorbitol gradient and cultured to late trophozoites following standard methods. Cells were washed three times in PBS and resuspended at 1% of haematocrit and 1-5% parasitaemia in 1% BSA-PBS solution. Five μl of plasma were added to 95 μl/well of erythrocyte suspension, incubated for 30 min at room temperature and stained for 30 min with 100 μl of polyclonal rabbit anti-human IgG (DakoCytomation) at 1/200. Subsequently, cells were incubated with 100 μl of AlexaFluor®-conjugated donkey anti-rabbit IgG (Invitrogen) diluted at 1/1000 and 10 mg/mL ethidium bromide for 30 min in darkness. Samples were washed three times with PBS-BSA between incubations. Data from 1000 positive events was acquired with a Becton-Dickinson FACSCalibur flow cytometer. Reactivity against IEs surface antigens was expressed as the difference between the mean fluorescence intensity (MFI) of IEs and the MFI of uninfected red blood cells.

### Cytokines and chemokines

Concentrations (pg/mL) of IL-12p70, IL-2, IFN-γ, IL-4, IL-5, IL-10, IL-8, IL-6, IL-1β, TNF, TNF-β and TGF-β1 in plasma were measured using fluorescent bead immunoassays (Human Th1/Th2 11plex and Human TGF-β1 FlowCytomix Simplex kits, Bender MedSystems, Austria) following manufacturer’s instructions. Beads fluorescence was acquired with a Becton-Dickinson FACSCanto II and analysed in FlowCytomix Pro2.2.1 software (Bender MedSystems). Concentration of each analyte was obtained by extrapolating fluorescence intensity to a 7-point dilution standard curve supplied by the manufacturer. Any value below the limits of detection was given a value of half the detection limit for that cytokine.

### Statistical methods

Prevalence of recognition of parasites and recombinant proteins by antibodies in children was considered positive if MFI or normalized OD values were above the mean of the negative controls plus three standard deviations for each antigen. Spearman’s rank correlations were performed to evaluate correlations between immunological parameters. Comparisons between matched case–control pairs for categorical variables were done using McNemar´s chi-squared, and reported as the number of pairs with a divergent result between SM and UM. Continuous variables were analysed using Sign test, and reported as the median difference between SM and UM values. Children were stratified by SM clinical presentations, and compared to their matched controls. Adjustment for multiple comparisons was done by Monte Carlo permutation tests with 1000 random permutations [39]. Associations between immune responses and parasite qPCR densities were assessed by multivariate linear regression with log-transformed parasite density as the outcome and log-transformed antibodies or cytokines as independent variables. All data collected were analysed using Stata version 10.0 (Stata Corporation). *P*-values <0.05 were considered statistically significant.

## Results

### Characteristics of the study participants

Among 142 children recruited, four tested negative for *P. falciparum* by qPCR and were excluded together with their matched pairs. Therefore, 134 children (67 case–control pairs) were finally analysed. The clinical characteristics of the patients are summarized in Table 1. Prostration, ARD, SA and MS were the most prevalent symptoms of severity, whereas hypoglycaemia and CM were only described in five and three children, respectively. Forty-four (65%) children presented with two or more SM criteria. The 23 cases with a single criterion of severity distributed as follows: 13 prostration, 6 MS, 3 SA and 1 ARD. Three SM patients died yielding a case-fatality rate of 2%, and two were transferred to Maputo Central Hospital. Parasite densities by qPCR were higher in SM cases than in paired controls, but this difference was not statistically significant (*P* = 0.087). Children with SM or UM did not differ in malnutrition, indirect clinical indicators of AIDS (oral candidiasis) or in the number of days with clinical symptoms prior to recruitment. Pre-treatment with an anti-malarial was only reported for one patient with SM. Previous history of SM was more common among children with SM (11 [16%]) than in children with UM (3 [4%]; divergent pairs = 13 [18%], *P* = 0.037).

**Table 1:** Characteristics of the study population

**Parameter**^*a*^	**Clinical groups**			
	**SM (N = 67)**	**UM (N = 67)**	**Matched comparison**^*c*^	***P***
**Demographic data**				
Age (months)	28 (16, 39)	29 (15, 38)	*-*	*-*
Males, n (%)	42 (63)	42 (63)	*-*	*-*
**Physical findings**				
Temperature (°C)	38.6 (37.9, 39.2)	38 (36.4, 39.5)	0.3 (−0.7, 2.2)	0.227
Weight (kg)	11.1 (8.9, 13)	10.9 (9.6, 12.8)	0 (−1.6, 1.2)	1.000
Malnutrition, n (%)	5 (8)	2 (3)	4 (6)	0.453
Oral candidiasis, n (%)	1 (1)	1 (1)	2 (3)	1.000
Hepatomegaly, n (%)	13 (19)	2 (3)	13 (19)	0.005
Splenomegaly, n (%)	35 (52)	12 (18)	35 (52)	<0.001
N° of days with symptoms^*b*^	1 (1, 3)	1 (1, 2)	0 (0, 1)	0.192
**Laboratory parameters**				
Parasitaemia by qPCR, (×10^3^/μL)	34.2 (7.5, 151.3)	13.3 (1.7, 51.6)	2.1 (−1.0, 21.8)	0.087
Parasitaemia by microscopy, (×10^3^/μL)	40.4 (17.3, 99.3)	34.7 (11.5, 71.3)	12.9 (−10.3, 58.3)	0.036
Multiplicity of infection	3 (2, 4)	3 (3, 4)	0 (−1, 2)	0.070
Packed cell volume (%)	26 (16, 31)	31 (28, 34)	−6 (−15, 0)	<0.001
Platelets (×10^9^/L)	117 (70, 185)	145 (92, 192)	−38 (−110, 43)	0.043
Glucose (mM)	5.8 (4.6, 7)	5.8 (5.3, 6.9)	−0.1 (−1.6, 1.2)	0.702
White blood cells (×10^9^/L)	10 (6.8, 12.7)	9.1 (7.2, 11.5)	0.7 (−2.8, 3.5)	0.110
Lymphocytes (%)	35.5 (24.7, 46)	40.6 (30.2, 50.8)	−7.6 (−19.0, 9.1)	0.008
Neutrophils (%)	56.9 (42.9, 67.1)	53.7 (36.9, 65.3)	5.8 (−5.1, 10.2)	0.043
Creatinin (U/L)	34 (30, 38)	34 (30, 39)	−1 (−6, 6)	0.470
Bilirubin (μM)	24 (14, 37)	13 (10, 21)	10 (−1, 23)	<0.001
ALT^*c*^ (U/L)	31.5 (19, 39)	28 (1, 36)	4 (−11, 15)	0.376
Lactate (mM)	3.8 (2.3, 5.1)	2.3 (1.9, 3.2)	1.0 (−0.3, 2.4)	0.001
**SM symptoms**				
Prostration, n (%)	50 (75)			
ARD ^*c*^, n (%)	34 (51)			
Severe anaemia, n (%)	22 (33)			
Multiple seizures, n (%)	19 (28)			
Hypoglycaemia, n (%)	5 (8)			
Cerebral malaria, n (%)	3 (5)			
N° of SM symptoms, n (%):				
One	23 (34)			
Two	27 (40)			
≥ three	17 (25)			

^*a*^ Presented as medians (inter-quartile range), except when indicated. qPCR, quantitative real-time polymerase chain reaction; ALT, alanine aminotransferase; ARD, acidosis and/or respiratory distress.

^*b*^ Fever, cough or vomits.

^*c*^ Matched comparison reports the median difference (inter-quartile range) between SM and UM values for continuous variables or the number (%) of divergent pairs for categorical variables. *P*-values calculated using McNemar´s or Sign test for matched pairs. All comparisons were corrected by the Monte Carlo permutation test (1000 random permutations).

### Antibodies and malaria severity

**Figure 1:**
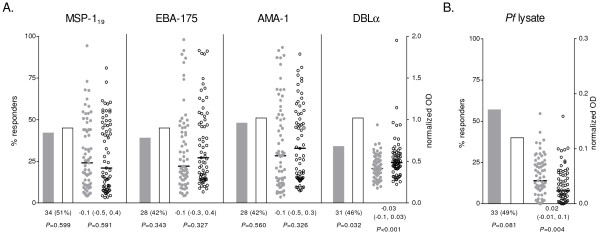
**IgG antibody responses to merozoite antigens (A) and*P. falciparum*(*Pf*) lysate (B).** Seroprevalence is represented by bars as the % of responders; plasma levels are represented by dot plots, with horizontal lines indicating median values. Data from children with SM is shown as shaded bars/dots; data from children with UM is shown as open bars/dots. Results of matched comparisons are reported in the x axis as the number (%) of divergent pairs for IgG seroprevalence, and the median difference (inter-quartile range) of IgG levels between SM and UM. *P*-values were calculated using McNemar’s or Sign test. All comparisons were corrected by the Monte Carlo permutation test (1000 random permutations). Thresholds for seroprevalence (OD): MSP-1_19_ = 0.560, EBA-175 = 0.628, AMA-1 = 0.578, DBLα = 0.489, *P. falciparum* lysate = 0.034. Median difference (inter-quartile range) of IgM levels against uninfected erythrocytes lysate between SM and UM = −0.01 [−0.03, 0.01], *P* = 0.175.

**Figure 2:**
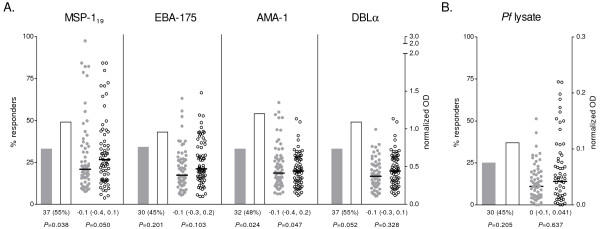
**IgM antibody responses to merozoite antigens (A) and*P. falciparum*(*Pf*) lysate (B).** Seroprevalence is represented by bars as the % of responders; plasma levels are represented by dot plots, with horizontal lines indicating median values. Data from children with SM is shown as shaded bars/dots; data from children with UM is shown as open bars/dots. Results of matched comparisons are reported in the x axis as the number (%) of divergent pairs for IgM seroprevalence, and the median difference (inter-quartile range) of IgM levels between SM and UM. *P*-values were calculated using McNemar’s or Sign test. All comparisons were corrected by the Monte Carlo permutation test (1000 random permutations). Thresholds for seroprevalence (OD): MSP-1_19_ = 0.605, EBA-175 = 0.514, AMA-1 = 0.554, DBLα = 0.444, *P. falciparum* lysate = 0.057. Median difference (inter-quartile range) of IgG levels against uninfected erythrocytes lysate between SM and UM = −0.02 [−0.2, 0.1], *P* = 1.000.

Prevalence and levels of IgG against DBLα were significantly lower in children with SM compared to their matched controls (*P =* 0.032 and *P* < 0.001, respectively), whereas no differences were found for IgG against merozoite antigens (Figure 1A). In contrast, IgG levels against *P. falciparum* lysate were significantly higher in children with SM than in matched controls (*P =* 0.004; Figure 1B). Recognition of antigens on the surface of IEs did not differ between SM and UM children for any of the parasites tested (Table 2). Overall, IgG prevalence was highest for R29 and E8B_CD36_ IEs, and lowest for CSA binding IEs (FCR3_CSA_) and placental isolates. A similar seroprevalence was found for IEs isolated from SM patients (MOZ1, MOZ2, MOZ4) or UM patients (MOZ3, MOZ5; Table 2). The number of IgM responders against MSP-1_19_ and AMA-1 was lower in SM (*P =* 0.038 and *P =* 0.024, respectively), and a similar but not significant trend was found for DBLα (Figure 2A). Likewise, anti-MSP-1_19_ and AMA-1 IgM levels were lower in SM compared to their UM pairs (*P =* 0.050 and *P =* 0.047, respectively). In general, different antibody responses correlated weakly (Additional file 1), with the exception of IgG against EBA-175 and MSP-1_19_ (Spearman’s *rho* = 0.8635, *P* < 0.001) and IgM against EBA-175 and AMA-1 (Spearman’s *rho* = 0.8595, *P* < 0.001).

**Table 2:** IgG antibody responses to infected erythrocytes surface antigens

**Parasites**^*a*^	**Seroprevalence**^*b*^**(N = 67 pairs)**				**IgG levels**^*c*^**(N = 67 pairs)**			
	**SM**	**UM**	**Matched comparison**	***P***	**SM**	**UM**	**Matched comparison**	***P***
R29	64 (96)	65 (97)	5 (7)	1.000	11.5 (8.7, 18.2)	9.9 (6.6, 20.2)	2.0 (−8.6, 9.1)	0.088
FCR3_CSA_	8 (12)	11 (16)	15 (22)	0.608	7.6 (6.6, 9.3)	7.7 (6.7, 9.2)	−0.3 (−2.3, 1.6)	0.609
E8B_CD36_	58 (87)	62 (93)	14 (21)	0.414	6.7 (4.2, 11.3)	6.9 (4.5, 10.7)	−0.8 (−4.3, 4.5)	0.636
ITG_ICAM1_	11 (16)	15 (22)	24 (36)	0.300	1.1 (0.1, 2.1)	1.3 (0.2, 2.5)	−0.4 (−2.5, 1.6)	0.345
MOZ1	17 (25)	18 (27)	25 (37)	0.666	28.0 (23.6, 31.8)	26.9 (24.5, 33.9)	0.01 (−5.5, 4.9)	1.000
MOZ2	40 (60)	36 (54)	34 (51)	0.367	1.8 (0.8, 5.9)	1.6 (0.8, 4.8)	0.7 (−2.3, 3.8)	0.334
MOZ3	32 (48)	30 (45)	34 (51)	0.611	2.6 (1.6, 4.1)	2.4 (1.3, 4.9)	0.2 (−2.3, 2.3)	0.650
MOZ4	47 (70)	44 (66)	27 (40)	0.704	5.3 (−1.4, 24.9)	3.3 (−1.4, 13.1)	2.8 (−11.9, 19.1)	0.329
MOZ5	51 (76)	56 (83)	24 (36)	0.293	88.0 (53.1, 115.4)	83.4 (62.3, 129.9)	−7.4 (−45.7, 35.6)	0.727
PLC1	0 (0)	3 (4)	3 (4)	0.255	58.3 (53.2, 64.8)	60.7 (51.9, 67.5)	−1.1 (−8.7, 7.3)	0.547
PLC2	0 (0)	3 (4)	3 (4)	0.255	19.8 (12.3, 24.2)	18.6 (10.1, 24.5)	−0.7 (−8.2, 8.9)	0.907

^*a*^ MOZ, isolates from Mozambican children; PLC, placental isolates from Mozambican women.

^*b*^ Presented as the number (%) of responders. Matched comparison reports the number (%) of divergent pairs. *P-*values calculated using McNemar’s test. Thresholds for seroprevalence (MFI): R29 = 5.1_,_ FCR3_CSA_ = 10.1, E8B_CD36_ = 2.6, ITG_ICAM_ = 2.7_,_ MOZ1 = 31.7, MOZ2 = 1.4, MOZ3 = 2.9, MOZ4 = 0.4, MOZ5 = 52.6, PLC1 = 107.6, PLC2 = 42.9.

^*c*^ Presented as medians (inter-quartile range) of MFI values. Matched comparison reports the median difference (inter-quartile range) of IgG levels between SM and UM. *P-*values calculated using Sign test.

All comparisons were corrected by the Monte Carlo permutation test (1000 random permutations).

A stratified analysis by SM clinical presentations showed that levels of IgG against DBLα were lower both for SA (*P =* 0.031), ARD (*P =* 0.015) and prostration (*P =* 0.002) compared to their respective matched controls (see Additional file 2). Similar to the main analysis, no differences were found for other recombinant antigens or for IgG against IEs surface (data not shown). Levels of IgM were significantly lower in children with SA, ARD and prostration for at least one of the antigens tested. Immune responses in children with CM and hypoglycaemia could not be assessed separately due to the low prevalence of these groups (Table 1).

A linear regression model including all antibody responses showed that IgG against DBLα was the only antibody independently associated to lower *P. falciparum* densities. The proportional reduction of parasite densities per a two-fold increment in IgG levels was 0.27 when both SM and UM children were included in the analysis ([95% confidence interval [CI] 0.08, 0.85], *P* = 0.026) and 0.11 including only SM cases ([95% confidence interval [CI] 0.02, 0.72], *P* = 0.022), but the effect was not observed among UM controls (1.34 [95% CI 0.28, 6.50], *P* = 0.712).

### Cytokines and chemokines and malaria severity

**Figure 3:**
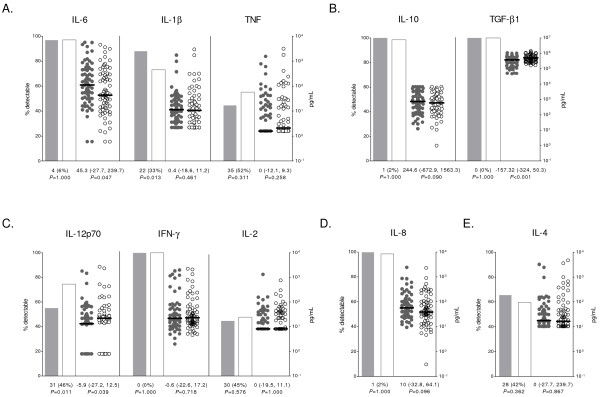
**Plasma concentrations of cytokines and chemokines.(A)** Pro-inflammatory cytokines, **(B)** immuno-regulatory/anti-inflammatory cytokines, **(C)** Th1 cytokines, **(D)** pro-inflammatory chemokine and **(E)** Th2 cytokine. Percentage of detectable cytokines/chemokines is represented by bars; concentrations are represented by dot plots, with horizontal lines indicating median values. Data from children with SM is shown as shaded bars/dots; data from children with UM is shown as open bars/dots. Results of matched comparisons are reported in the x axis as the number (%) of divergent pairs for cytokine/chemokine prevalence, and the median difference (inter-quartile range) of cytokine/chemokine concentrations between SM and UM (in ng/mL for TGF-β1). *P*-values were calculated using McNemar’s or Sign test. All comparisons were corrected by the Monte Carlo permutation test (1000 random permutations).

Children with SM had significantly higher IL-6 concentrations in plasma than children with UM (*P* = 0.047, Figure 3A). IL-1β was more frequently detected in children with SM (*P =* 0.013), although no significant differences were found for IL-1β concentrations. Conversely, children with SM had lower levels of TGF-β1 and IL-12 (*P* < 0.001 and *P* = 0.039, respectively; Figure 3B and 3C), and lower prevalence of detectable IL-12 concentrations than matched UM controls (*P =* 0.011; Figure 3C). IL-5 and TNF-β were excluded from the statistical analysis since levels in most samples were below the limit of detection (109/134 [81%] for IL-5 and 132/134 [99%] for TNF-β). Overall, there were weak or moderate correlations within cytokine responses (Additional file 3), with the exception of IL-6 and IL-10 (Spearman’s *rho* = 0.8682, *P* < 0.001). No significant correlations were found between antibodies and cytokines.

Stratification by severe clinical presentations showed that children with ARD had higher levels of IL-10 (*P =* 0.032) and IL-6 (*P =* 0.002) and lower levels of TGF-β1 (*P =* 0.002) compared to their controls (Additional file 2). TGF-β1 concentrations were also reduced in the prostration group (*P* = 0.007)*.*

IL-12 concentrations were associated with a reduction of parasite densities when all children were included in the analysis (0.69 [95% CI 0.54, 0.90], *P* = 0.006) and including only children with SM (0.62 [95% CI 0.44, 0.89], *P* = 0.010), but the association was not found in children with UM (0.88 [95% CI 0.58, 1.33], *P* = 0.528). On the other hand, higher concentrations of IL-10 were associated with an increase in parasite densities in all groups (all children: 2.44 [95% CI 1.58, 3.77], *P* < 0.001; SM cases: 2.59 [95% CI 1.37, 4.92], *P* = 0.004; UM controls: 2.62 [95% CI 1.38, 5.01], *P* = 0.004).

## Discussion

Results of this case–control study show that SM in Mozambican children under five years of age is associated with low IgM responses to blood stage antigens and low IgG responses to a rosetting DBLα domain [33], as well as with high levels of pro-inflammatory IL-6 and low levels of TGF-β1 and IL-12. In contrast to previous reports, this study did not find differences in IgG responses against MSP-1_19_[7], AMA-1 [40] or IEs surface antigens [10], possibly due to different definitions of SM clinical presentations and levels of endemicity in the study settings.

Although the observational approach of the study does not allow determining if lower levels of antibodies are the cause or the consequence of a severe infection, the fact that immunoglobulin reduction was not uniform strongly argues against a general impairment of immune response as a consequence of SM. The reduction of anti-DBLα IgG suggests that DBLα domains involved in rosetting are an important target of immunity against SM, both for presentations such as ARD, prostration and SA (and being the lack of association with MS probably attributable to low sample size). IgG antibodies to several DBLα domains have already been linked to natural acquisition of immunity [33,41] and to reduced risk of clinical malaria [42]. The finding is further supported by a negative association between IgG to DBLα and parasite densities. Antibodies against this *Pf*EMP1 domain may be able to reduce parasite densities through disruption of rosettes, a mechanism that has been associated with protection from SM [43]. Conversely, no difference was found for IgG against surface antigens of R29 IEs, the parasite from which DBLα was cloned [33], suggesting that other R29 *var1Pf*EMP1 domains and other antigens on the surface of IEs may be masking epitope-specific DBLα responses. Despite the absence of differences between clinical groups, prevalence of recognition of R29 surface antigens was high. R29 predominantly expresses a group A *Pf*EMP1 [44], to which antibodies are rapidly acquired during early childhood [41,45]. On the other hand, no significant recognition of the pregnancy-specific CSA-binding parasites was detected, in contrast with a recent study that suggests recognition of VAR2CSA DBL5ϵ domain in young children [45].

This study also reports a reduction of IgM responses to blood stage antigens in children with SM compared to UM, in agreement with findings from previous studies [12-14]. IgM levels have been associated with reduced risk of malaria in children and adolescents [46,47] and negatively correlated with parasite densities [46], suggesting that IgM are capable of controlling *Plasmodium* infection through early limitation of parasite growth [48] or through blocking of pro-inflammatory TNF production [49].

The differences in antibody levels between children with SM and UM observed in this study were not attributable to a lower exposure to *P. falciparum* in SM cases, since children with SM had similar levels of IgM and even higher IgG against total *P. falciparum* antigens compared to children with UM. Moreover, differences could not be explained by a longer progression of the disease in SM cases because the number of days with symptoms of malaria prior to enrolment was similar to that in the UM group. Importantly, a higher number of previous SM episodes were reported among cases. This observation may indicate that host genetic factors can predispose children to develop SM [50] or that children in the SM group were subject to a higher *in utero* exposure to *P. falciparum* antigens, producing immunological tolerance and increasing susceptibility to subsequent malaria infections [51]. Also, an episode of SM may render children more susceptible to future severe episodes, maybe through an expansion of exhausted T cells or atypical B cells [52,53] which may impair the development of an adequate protective immune response.

SM was also found to be associated with alterations of the fine balance between pro-inflammatory and anti-inflammatory cytokines [22]. Children with SM showed higher levels of pro-inflammatory IL-6 [16,54], had more detectable levels of IL-1β and insufficient immuno-regulatory TGF-β1, which has been shown to counterbalance the pro-inflammatory response [22]. Although levels of immuno-regulatory IL-10 were significantly increased in children with ARD [55] this response did not seem to be sufficient to control the IL-6 imbalance. As previously reported by Lyke *et al.* in Mali [54], TNF did not differ between SM and UM in Mozambican children, an observation that is in contrast to other studies suggesting that TNF is the major cytokine involved in the pro-inflammatory response in SM [56,57]. It is possible that IL-6 and IL-10 levels reached by SM children at admission had already down regulated initial TNF pro-inflammatory response [19,20]. Children with SM were also characterized by lower levels of IL-12 [22,57], a cytokine previously associated with anti-malarial immunity [15,17,22]. Consistently, a negative association between IL-12 concentrations and parasite densities was found. Haemozoin phagocytosis by monocytes but also IL-10 may be acting as IL-12 inhibitors in SM [57,58]. Overall, this study suggests that children with SM may have an immature immune system typical of early childhood, consisting in a greater dependence on innate immune responses for protection against infections [59], lower Th1-polarizing cytokine responses (i.e. IL-12) [60,61] and a bias of Toll-Like receptor-mediated responses towards acute phase (i.e. IL-1β and IL-6) and IL-10 responses [60]. Therefore, an immune system still under development, together with an insufficient or impaired antibody response at this age, may limit the ability to contain infection, lead to an exacerbated inflammatory response and increase the risk of developing SM.

The present study is limited by a number of factors. First, antibodies and cytokines measured in peripheral blood at a single time point may not reflect the course of infection or the response in specific organs where parasite sequestration is occurring. Second, it was not possible to control for the effects that co-infection with HIV or helminthes may have on anti-malarial immunity [62,63]. Nevertheless, clinical data suggests that AIDS was infrequent in patients from the present study. Finally, the high degree of overlap in severe symptoms, which is otherwise common in endemic areas [64], together with the limited sample size of clinical groups, may have hampered the identification of immunological correlates that are particular to a clinical form of SM.

## Conclusions

These data supports a role for specific IgM to *P. falciparum* and IgG to rosetting *Pf*EMP1 domains in controlling SM. Also, imbalanced pro-inflammatory cytokine responses may exacerbate the severity of infection in children. This information is of relevance to understand how immunity to SM is acquired in early childhood and also for the design of strategies to control life-threatening malaria. In particular, subunit vaccines containing DBLα epitopes from rosetting *Pf*EMP1 might potentially elicit functional antibodies that reduce the risk of SM in children. Prospective cohort studies assessing IgG isotypes and functionality of antibodies, together with detailed phenotype analysis of cytokine-producing cells will help to understand the mechanisms underlying natural acquisition of immunity to malaria severe disease.

## Abbreviations

AMA: apical membrane antigen; ARD: acidosis and/or respiratory distress; CM: cerebral malaria; DBL: Duffy binding like; EBA: erythrocyte binding antigen; IEs: infected erythrocytes; IFN: interferon; IgG: immunoglobulin G; IgM: immunoglobulin M; IL: interleukin; MFI: mean fluorescence intensity; MOI: multiplicity of infection; MS: multiple seizures; MSP: merozoite surface protein; OD: optical density; PfEMP: Plasmodium falciparum erythrocyte membrane protein; SA: severe anaemia; SM: severe malaria; TNF: tumor necrosis factor; TGF: transforming growth factor; UM: uncomplicated malaria.

## Competing interests

Authors declare that they have no competing interests.

## Author’s contributions

ERV and GM carried out the antibody and cytokine measurements, the analysis and interpretation of data and wrote the manuscript. QB and SM performed the clinical diagnosis and management of malaria patients. RA coordinated data collection and sample processing. LP contributed to sample processing and antibody measurements. LQ designed statistical analysis and helped to draft the manuscript. CC produced the recombinant proteins. CM and PA contributed to the study design and drafting of the manuscript. AM and CD conceived and coordinated the study, participated in the analysis and interpretation of the data and contributed to the preparation of the manuscript. All authors read and approved the final manuscript.

## Supplementary Material

Additional file 1 Spearman’s rank correlation coefficients between IgG and IgM levels in the study population. * *P* < 0.05. Click here for file

Additional file 2 Matched comparisons of antibody levels and cytokine and chemokine concentrations in children with different severe malaria clinical presentations. Click here for file

Additional file 3 Spearman’s rank correlation coefficients between cytokine and chemokine concentrations in the study population. * *P* < 0.05. Click here for file

## References

[B1] WHO World Malaria Report 2011 Geneva: World Health Organization

[B2] Miller LH Baruch DI Marsh K Doumbo OK The pathogenic basis of malaria Nature 2002 415 673 679 10.1038/415673a 11832955

[B3] Doolan DL Dobaño C Baird JK Acquired immunity to malaria Clin Microbiol Rev 2009 22 13 36 10.1128/CMR.00025-08 19136431 PMC2620631

[B4] Cohen S McGregor IA Carrington S Gamma-globulin and acquired immunity to human malaria Nature 1961 192 733 737 10.1038/192733a0 13880318

[B5] Carlson J Helmby H Hill AV Brewster D Greenwood BM Wahlgren M Human cerebral malaria: association with erythrocyte rosetting and lack of anti-rosetting antibodies Lancet 1990 336 1457 1460 10.1016/0140-6736(90)93174-N 1979090

[B6] Mo M Lee HC Kotaka M Niang M Gao X Iyer JK Lescar J Preiser P The C-terminal segment of the cysteine-rich interdomain of Plasmodium falciparum erythrocyte membrane protein 1 determines CD36 binding and elicits antibodies that inhibit adhesion of parasite-infected erythrocytes Infect Immun 2008 76 1837 1847 10.1128/IAI.00480-07 18299339 PMC2346709

[B7] Dobaño C Rogerson SJ Mackinnon MJ Cavanagh DR Taylor TE Molyneux ME McBride JS Differential antibody responses to Plasmodium falciparum merozoite proteins in Malawian children with severe malaria J Infect Dis 2008 197 766 774 10.1086/527490 18260767

[B8] Okech B Mujuzi G Ogwal A Shirai H Horii T Egwang TG High titers of IgG antibodies against Plasmodium falciparum serine repeat antigen 5 (SERA5) are associated with protection against severe malaria in Ugandan children AmJTrop Med Hyg 2006 74 191 197 16474069

[B9] Erunkulu OA Hill AV Kwiatkowski DP Todd JE Iqbal J Berzins K Riley EM Greenwood BM Severe malaria in Gambian children is not due to lack of previous exposure to malaria Clin Exp Immunol 1992 89 296 300 1638773 10.1111/j.1365-2249.1992.tb06948.x PMC1554452

[B10] Tebo AE Kremsner PG Piper KP Luty AJ Low antibody responses to variant surface antigens of Plasmodium falciparum are associated with severe malaria and increased susceptibility to malaria attacks in Gabonese children AmJTrop Med Hyg 2002 67 597 603 10.4269/ajtmh.2002.67.597 12518849

[B11] Iriemenam NC Khirelsied AH Nasr A ElGhazali G Giha HA Elhassan AETM Agab-Aldour AA Montgomery SM Anders RF Theisen M Troye-Blomberg M Elbashir MI Berzins K Antibody responses to a panel of Plasmodium falciparum malaria blood-stage antigens in relation to clinical disease outcome in Sudan Vaccine 2009 27 62 71 10.1016/j.vaccine.2008.10.025 18977268

[B12] Luty AJ Ulbert S Lell B Lehman L Schmidt-Ott R Luckner D Greve B Matousek P Schmid D Herbich K Dubois B Deloron P Kremsner PG Antibody responses to Plasmodium falciparum: evolution according to the severity of a prior clinical episode and association with subsequent reinfection AmJTrop Med Hyg 2000 62 566 572 10.4269/ajtmh.2000.62.566 11289665

[B13] Brasseur P Ballet JJ Druilhe P Impairment of Plasmodium falciparum-specific antibody response in severe malaria J Clin Microbiol 1990 28 265 268 2179259 10.1128/jcm.28.2.265-268.1990 PMC269588

[B14] Fribourg-Blanc A Druilhe P Brasseur P Rhodes-Feuillette A Ballet JJ Tharavanij S Immunological evaluation of cell-mediated and humoral immunity in Thai patients with cerebral and non cerebral Plasmodium falciparum malaria: II. Evolution of serum levels of immunoglobulins, antimalarial antibodies, complement fractions and alpha interferon Southeast Asian J Trop Med Public Health 1985 16 307 313 3906923

[B15] Sedegah M Finkelman F Hoffman SL Interleukin 12 induction of interferon gamma-dependent protection against malaria Proc Natl Acad Sci U S A 1994 91 10700 10702 10.1073/pnas.91.22.10700 7938013 PMC45089

[B16] Kremsner PG Winkler S Brandts C Wildling E Jenne L Graninger W Prada J Bienzle U Juillard P Grau GE Prediction of accelerated cure in Plasmodium falciparum malaria by the elevated capacity of tumor necrosis factor production AmJTrop Med Hyg 1995 53 532 538 10.4269/ajtmh.1995.53.532 7485713

[B17] Hoffman SL Crutcher JM Puri SK Ansari AA Villinger F Franke ED Singh PP Finkelman F Gately MK Dutta GP Sedegah M Sterile protection of monkeys against malaria after administration of interleukin-12 Nat Med 1997 3 80 83 10.1038/nm0197-80 8986746

[B18] Mackintosh CL Beeson JG Marsh K Clinical features and pathogenesis of severe malaria Trends Parasitol 2004 20 597 603 10.1016/j.pt.2004.09.006 15522670

[B19] Eckwalanga M Marussig M Tavares MD Bouanga JC Hulier E Pavlovitch JH Minoprio P Portnoi D Renia L Mazier D Murine AIDS protects mice against experimental cerebral malaria: down-regulation by interleukin 10 of a T-helper type 1 CD4+ cell-mediated pathology Proc Natl Acad Sci U S A 1994 91 8097 8101 10.1073/pnas.91.17.8097 8058763 PMC44552

[B20] Ho M Sexton MM Tongtawe P Looareesuwan S Suntharasamai P Webster HK Interleukin-10 inhibits tumor necrosis factor production but not antigen-specific lymphoproliferation in acute Plasmodium falciparum malaria J Infect Dis 1995 172 838 844 10.1093/infdis/172.3.838 7658079

[B21] Omer FM Riley EM Transforming growth factor beta production is inversely correlated with severity of murine malaria infection J Exp Med 1998 188 39 48 10.1084/jem.188.1.39 9653082 PMC2525539

[B22] Perkins DJ Weinberg JB Kremsner PG Reduced interleukin-12 and transforming growth factor-beta1 in severe childhood malaria: relationship of cytokine balance with disease severity J Infect Dis 2000 182 988 992 10.1086/315762 10950804

[B23] Bassat Q Guinovart C Sigauque B Aide P Sacarlal J Nhampossa T Bardaji A Nhacolo A Macete E Mandomando I Aponte JJ Menéndez C Alonso P Malaria in rural Mozambique. Part II: children admitted to hospital Malar J 2008 7 37 10.1186/1475-2875-7-37 18302771 PMC2275288

[B24] Alonso P Saúte F Aponte JJ Gómez-Olivé FX Nhacolo A Thomson R Macete E Abacassamo F Ventura PJ Bosch X Menéndez C Dgedge M Manhiça DSS, Mozambique. Population, Health and Survival at INDEPTH Sites. Volume 1 2001 Ottawa: INDEPTH Network 189 195

[B25] Mayor A Hafiz A Bassat Q Rovira-Vallbona E Sanz S Machevo S Aguilar R Cisteró P Sigaúque B Menéndez C Alonso PL Chitnis CE Association of severe malaria outcomes with platelet-mediated clumping and adhesion to a novel host receptor PLoS One 2011 6 e19422 10.1371/journal.pone.0019422 21559373 PMC3084855

[B26] Saute F Aponte J Almeda J Ascaso C Abellana R Vaz N Dgedge M Alonso P Malaria in southern Mozambique: malariometric indicators and malaria case definition in Manhica district Trans R Soc Trop Med Hyg 2003 97 661 666 10.1016/S0035-9203(03)80098-6 16117958

[B27] Alonso PL Sacarlal J Aponte JJ Leach A Macete E Milman J Mandomando I Spiessens B Guinovart C Espasa M Bassat Q Aide P Ofori-Anyinam O Navia MM Corachan S Ceuppens M Dubois MC Demoitie MA Dubovsky F Menendez C Tornieporth N Ballou WR Thompson R Cohen J Efficacy of the RTS, S/AS02A vaccine against Plasmodium falciparum infection and disease in young African children: randomised controlled trial Lancet 2004 364 1411 1420 10.1016/S0140-6736(04)17223-1 15488216

[B28] Hermsen CC Telgt DSC Linders EHP van de Locht LATF Eling WMC Mensink EJBM Sauerwein RW Detection of Plasmodium falciparum malaria parasites in vivo by real-time quantitative PCR Mol Biochem Parasitol 2001 118 247 251 10.1016/S0166-6851(01)00379-6 11738714

[B29] Snounou G Zhu X Siripoon N Jarra W Thaithong S Brown KN Viriyakosol S Biased distribution of msp1 and msp2 allelic variants in Plasmodium falciparum populations in Thailand Trans R Soc Trop Med Hyg 1999 93 369 374 10.1016/S0035-9203(99)90120-7 10674079

[B30] Mazumdar S Sachdeva S Chauhan VS Yazdani SS Identification of cultivation condition to produce correctly folded form of a malaria vaccine based on Plasmodium falciparum merozoite surface protein-1 in Escherichia coli Bioprocess Biosyst Eng 2010 33 719 730 10.1007/s00449-009-0394-x 19921275

[B31] Pandey KC Singh S Pattnaik P Pillai CR Pillai U Lynn A Jain SK Chitnis CE Bacterially expressed and refolded receptor binding domain of Plasmodium falciparum EBA-175 elicits invasion inhibitory antibodies Mol Biochem Parasitol 2002 123 23 33 10.1016/S0166-6851(02)00122-6 12165386

[B32] Kocken CH Withers-Martinez C Dubbeld MA van der Wel A Hackett F Valderrama A Blackman MJ Thomas AW High-level expression of the malaria blood-stage vaccine candidate Plasmodium falciparum apical membrane antigen 1 and induction of antibodies that inhibit erythrocyte invasion Infect Immun 2002 70 4471 4476 10.1128/IAI.70.8.4471-4476.2002 12117958 PMC128198

[B33] Mayor A Rovira-Vallbona E Srivastava A Sharma SK Pati SS Puyol L Quinto L Bassat Q Machevo S Mandomando I Chauhan VS Alonso PL Chitnis CE Functional and immunological characterization of a Duffy binding-like alpha domain from Plasmodium falciparum erythrocyte membrane protein 1 that mediates rosetting Infect Immun 2009 77 3857 3863 10.1128/IAI.00049-09 19546191 PMC2738012

[B34] Mayor A Rovira-Vallbona E Machevo S Bassat Q Aguilar R Quinto L Jimenez A Sigauque B Dobano C Kumar S Singh B Gupta P Chauhan VS Chitnis CE Alonso PL Menéndez C Parity and placental infection affect antibody responses against Plasmodium falciparum during pregnancy Infect Immun 2011 79 1654 1659 10.1128/IAI.01000-10 21300778 PMC3067565

[B35] Rowe JA Moulds JM Newbold CI Miller LH P. falciparum rosetting mediated by a parasite-variant erythrocyte membrane protein and complement-receptor 1 Nature 1997 388 292 295 10.1038/40888 9230440

[B36] Ockenhouse CF Ho M Tandon NN Van Seventer GA Shaw S White NJ Jamieson GA Chulay JD Webster HK Molecular basis of sequestration in severe and uncomplicated Plasmodium falciparum malaria: differential adhesion of infected erythrocytes to CD36 and ICAM-1 J Infect Dis 1991 164 163 169 10.1093/infdis/164.1.163 1711552

[B37] Viebig NK Gamain B Scheidig C Lepolard C Przyborski J Lanzer M Gysin J Scherf A A single member of the Plasmodium falciparum var multigene family determines cytoadhesion to the placental receptor chondroitin sulphate A EMBO Rep 2005 6 775 781 10.1038/sj.embor.7400466 16025132 PMC1369142

[B38] Beeson JG Mann EJ Elliott SR Lema VM Tadesse E Molyneux ME Brown GV Rogerson SJ Antibodies to variant surface antigens of Plasmodium falciparum-infected erythrocytes and adhesion inhibitory antibodies are associated with placental malaria and have overlapping and distinct targets J Infect Dis 2004 189 540 551 10.1086/381186 14745713 PMC2613478

[B39] Reitmeir P Wassmer G Resampling-based methods for the analysis of multiple endpoints in clinical trials Stat Med 1999 18 3453 3462 10.1002/(SICI)1097-0258(19991230)18:24<3453::AID-SIM283>3.0.CO;2-Z 10611618

[B40] Iriemenam NC Okafor CM Balogun HA Ayede I Omosun Y Persson JO Hagstedt M Anumudu CI Nwuba RI Troye-Blomberg M Berzins K Cytokine profiles and antibody responses to Plasmodium falciparum malaria infection in individuals living in Ibadan, southwest Nigeria Afr Health Sci 2009 9 66 74 19652739 PMC2707050

[B41] Cham GK Turner L Lusingu J Vestergaard L Mmbando BP Kurtis JD Jensen ATR Salanti A Lavstsen T Theander TG Sequential, Ordered Acquisition of Antibodies to Plasmodium falciparum Erythrocyte Membrane Protein 1 Domains J Immunol 2009 183 3356 3363 10.4049/jimmunol.0901331 19675168

[B42] Mackintosh CL Christodoulou Z Mwangi TW Kortok M Pinches R Williams TN Marsh K Newbold CI Acquisition of naturally occurring antibody responses to recombinant protein domains of Plasmodium falciparum erythrocyte membrane protein 1 Malar J 2008 7 155 10.1186/1475-2875-7-155 18706102 PMC2533674

[B43] Carlson J Nash GB Gabutti V al-Yaman F, Wahlgren M: Natural protection against severe Plasmodium falciparum malaria due to impaired rosette formation Blood 1994 84 3909 3914 7949147

[B44] Rovira-Vallbona E Dobaño C Bardají A Cisteró P Romagosa C Serra-Casas E Quinto L Bassat Q Sigaúque B Alonso PL Ordi J Menéndez C Mayor A Transcription of var genes other than var2csa in Plasmodium falciparum parasites infecting Mozambican pregnant women J Infect Dis 2011 204 27 35 10.1093/infdis/jir217 21628655 PMC3307158

[B45] Oleinikov AV Voronkova VV Frye IT Amos E Morrison R Fried M Duffy PE A Plasma Survey Using 38 PfEMP1 Domains Reveals Frequent Recognition of the Plasmodium falciparum Antigen VAR2CSA among Young Tanzanian Children PLoS One 2012 7 e31011 10.1371/journal.pone.0031011 22295123 PMC3266279

[B46] Boudin C Chumpitazi B Dziegiel M Peyron F Picot S Hogh B Ambroise-Thomas P Possible role of specific immunoglobulin M antibodies to Plasmodium falciparum antigens in immunoprotection of humans living in a hyperendemic area, Burkina Faso J Clin Microbiol 1993 31 636 641 8458956 10.1128/jcm.31.3.636-641.1993 PMC262833

[B47] Dodoo D Aikins A Kusi KA Lamptey H Remarque E Milligan P Bosomprah S Chilengi R Osei YD Akanmori BD Theisen M Cohort study of the association of antibody levels to AMA1, MSP119, MSP3 and GLURP with protection from clinical malaria in Ghanaian children Malar J 2008 7 142 10.1186/1475-2875-7-142 18664257 PMC2529305

[B48] Couper KN Phillips RS Brombacher F Alexander J Parasite-specific IgM plays a significant role in the protective immune response to asexual erythrocytic stage Plasmodium chabaudi AS infection Parasite Immunol 2005 27 171 180 10.1111/j.1365-3024.2005.00760.x 15987340

[B49] Bate CA Taverne J Dave A Playfair JH Malaria exoantigens induce T-independent antibody that blocks their ability to induce TNF Immunology 1990 70 315 320 2379940 PMC1384159

[B50] Driss A Hibbert JM Wilson NO Iqbal SA Adamkiewicz TV Stiles JK Genetic polymorphisms linked to susceptibility to malaria Malar J 2011 10 271 10.1186/1475-2875-10-271 21929748 PMC3184115

[B51] Malhotra I Dent A Mungai P Wamachi A Ouma JH Narum DL Muchiri E Tisch DJ King CL Can prenatal malaria exposure produce an immune tolerant phenotype? A prospective birth cohort study in Kenya PLoS Med 2009 6 e1000116 10.1371/journal.pmed.1000116 19636353 PMC2707618

[B52] Butler NS Moebius J Pewe LL Traore B Doumbo OK Tygrett LT Waldschmidt TJ Crompton PD Harty JT Therapeutic blockade of PD-L1 and LAG-3 rapidly clears established blood-stage Plasmodium infection Nat Immunol 2011 13 188 195 10.1038/ni.2180 22157630 PMC3262959

[B53] Weiss GE Crompton PD Li S Walsh LA Moir S Traore B Kayentao K Ongoiba A Doumbo OK Pierce SK Atypical memory B cells are greatly expanded in individuals living in a malaria-endemic area J Immunol 2009 183 2176 2182 10.4049/jimmunol.0901297 19592645 PMC2713793

[B54] Lyke KE Burges R Cissoko Y Sangare L Dao M Diarra I Kone A Harley R Plowe CV Doumbo OK Sztein MB Serum levels of the proinflammatory cytokines interleukin-1 beta (IL-1beta), IL-6, IL-8, IL-10, tumor necrosis factor alpha, and IL-12(p70) in Malian children with severe Plasmodium falciparum malaria and matched uncomplicated malaria or healthy controls Infect Immun 2004 72 5630 5637 10.1128/IAI.72.10.5630-5637.2004 15385460 PMC517593

[B55] Awandare GA Goka B Boeuf P Tetteh JK Kurtzhals JA Behr C Akanmori BD Increased levels of inflammatory mediators in children with severe Plasmodium falciparum malaria with respiratory distress J Infect Dis 2006 194 1438 1446 10.1086/508547 17054074

[B56] Grau GE Taylor TE Molyneux ME Wirima JJ Vassalli P Hommel M Lambert PH Tumor necrosis factor and disease severity in children with falciparum malaria N Engl J Med 1989 320 1586 1591 10.1056/NEJM198906153202404 2657427

[B57] Luty AJ Perkins DJ Lell B Schmidt-Ott R Lehman LG Luckner D Greve B Matousek P Herbich K Schmid D Weinberg JB Kremsner PG Low interleukin-12 activity in severe Plasmodium falciparum malaria Infect Immun 2000 68 3909 3915 10.1128/IAI.68.7.3909-3915.2000 10858202 PMC101666

[B58] Keller CC Yamo O Ouma C Ong'echa JM, Ounah D, Hittner JB, Vulule JM, Perkins DJ: Acquisition of hemozoin by monocytes down-regulates interleukin-12 p40 (IL-12p40) transcripts and circulating IL-12p70 through an IL-10-dependent mechanism: in vivo and in vitro findings in severe malarial anemia Infect Immun 2006 74 5249 5260 10.1128/IAI.00843-06 16926419 PMC1594872

[B59] PrabhuDas M Adkins B Gans H King C Levy O Ramilo O Siegrist CA Challenges in infant immunity: implications for responses to infection and vaccines Nat Immunol 2011 12 189 194 21321588 10.1038/ni0311-189

[B60] Burl S Townend J Njie-Jobe J Cox M Adetifa UJ Touray E Philbin VJ Mancuso C Kampmann B Whittle H Jaye A Flanagan KL Levy O Age-dependent maturation of Toll-like receptor-mediated cytokine responses in Gambian infants PLoS One 2011 6 e18185 10.1371/journal.pone.0018185 21533209 PMC3076452

[B61] Adkins B Leclerc C Marshall-Clarke S Neonatal adaptive immunity comes of age Nat Rev Immunol 2004 4 553 564 10.1038/nri1394 15229474

[B62] Courtin D Djilali-Saiah A Milet J Soulard V Gaye O Migot-Nabias F Sauerwein R Garcia A Luty AJ Schistosoma haematobium infection affects Plasmodium falciparum-specific IgG responses associated with protection against malaria Parasite Immunol 2011 33 124 131 10.1111/j.1365-3024.2010.01267.x 21226725

[B63] Flateau C Le Loup G Pialoux G Consequences of HIV infection on malaria and therapeutic implications: a systematic review Lancet Infect Dis 2011 11 541 556 10.1016/S1473-3099(11)70031-7 21700241

[B64] Maitland K Marsh K Pathophysiology of severe malaria in children Acta Trop 2004 90 131 140 10.1016/j.actatropica.2003.11.010 15177139

