# Supplementary material for: Low antibodies against Plasmodium falciparum and imbalanced pro-inflammatory cytokines are associated with severe malaria in Mozambican children: a case–control study
Source: Malar J. 2012 May 30;11:181. doi: 10.1186/1475-2875-11-181 (PMC3464173; doi:10.1186/1475-2875-11-181)
Supplement: Additional file 1 — Spearman’s rank correlation coefficients between IgG and IgM levels in the study population. * P < 0.05. [file 1475-2875-11-181-S1.pdf]

Additional file 1. Spearman's rank correlation coefficients between IgG and IgM levels in the study population. * *P* <0.05.

		IgG																IgM			
		MSP-1	EBA-175	AMA-1	DBLα	*Pf* lysate	R29	$E8B_{CD36}$	ITG_{ICAM1}	$FCR3_{CSA}$	MOZ1	MOZ2	MOZ3	MOZ4	MOZ5	PLC1	PLC2	MSP-1	EBA-175	AMA-1	DBLα
IgG	EBA-175	0.8635 *																			
IgG	AMA-1	0.4341 *	0.5262 *																		
IgG	DBLα	0.1080	0.2275	0.0719																	
IgG	*Pf* lysate	0.5562 *	0.5795 *	0.5035 *	-0.0073																
IgG	R29	-0.0261	0.0350	0.3348 *	0.1236	0.2422															
IgG	$E8B_{CD36}$	-0.1781	-0.0721	0.1648	0.0861	0.0166	0.2542														
IgG	ITG_{ICAM1}	-0.3889 *	-0.3390 *	-0.0477	-0.1157	-0.2626	0.3192 *	0.2050													
IgG	$FCR3_{CSA}$	-0.1548	-0.1557	-0.0233	-0.1209	-0.0922	0.0854	0.0642	0.2744												
IgG	MOZ1	-0.0692	0.0009	0.1781	0.2182	0.1734	0.4140 *	0.0752	0.0562	-0.0576											
IgG	MOZ2	-0.3227 *	-0.2442	0.2657	0.0271	-0.0139	0.4975 *	0.2734	0.4696 *	0.0104	0.4620 *										
IgG	MOZ3	-0.1210	0.0098	0.2775	0.1525	0.1606	0.5068 *	0.0873	0.3338 *	-0.0076	0.5745 *	0.5789 *									
IgG	MOZ4	-0.0604	0.0076	0.3050	0.0873	0.2204	0.5330 *	0.0937	0.2045	-0.0224	0.6257 *	0.6094 *	0.6015 *								
IgG	MOZ5	-0.0460	0.0858	0.3002	0.3231 *	0.1628	0.5638 *	0.0814	0.2051	-0.0736	0.5856 *	0.5353 *	0.5153 *	0.5614 *							
IgG	PLC1	0.0542	0.1043	0.1236	0.3303 *	0.1115	0.2294	0.1284	-0.0587	0.0437	0.3638 *	0.1952	0.1046	0.2461	0.3017						
IgG	PLC2	0.0058	-0.0758	0.0645	-0.2979	0.1069	0.0715	-0.1459	0.0774	0.0764	0.1045	0.0887	0.1473	0.1778	-0.0813	0.0846					
IgM	MSP-1	0.4029 *	0.3020	0.0704	0.1570	0.0552	-0.2903	-0.1997	-0.1923	-0.0518	0.0187	-0.2317	-0.1053	-0.0782	-0.0115	-0.0178	-0.0785				
IgM	EBA-175	0.4345 *	0.4645 *	0.2353	0.1583	0.2778	-0.0803	-0.1384	-0.1413	-0.1072	0.0995	-0.1256	0.0188	-0.0215	0.1928	0.1980	-0.0752	0.5755 *			
IgM	AMA-1	0.4210 *	0.4355 *	0.2040	0.2307	0.1839	-0.1728	-0.1118	-0.1676	-0.0524	0.0744	-0.1846	-0.0601	-0.0614	0.1215	0.1498	-0.1334	0.7270 *	0.8595 *		
IgM	DBLα	0.3368 *	0.2876	0.1144	0.0759	0.1171	-0.1603	-0.1069	-0.0339	0.0571	0.0301	-0.1253	-0.0945	-0.0357	-0.0127	0.1590	0.0858	0.4817 *	0.5857 *	0.5933 *	
IgM	*Pf* lysate	0.3261 *	0.3034	0.1078	0.0062	0.3219 *	0.0283	-0.1140	-0.1151	-0.0831	0.0746	-0.1242	0.1345	0.0392	0.1194	-0.0436	0.0811	0.4284 *	0.4173 *	0.4401 *	0.1535
