# Supplementary material for: Low antibodies against Plasmodium falciparum and imbalanced pro-inflammatory cytokines are associated with severe malaria in Mozambican children: a case–control study
Source: Malar J. 2012 May 30;11:181. doi: 10.1186/1475-2875-11-181 (PMC3464173; doi:10.1186/1475-2875-11-181)
Supplement: Additional file 2 — Matched comparisons of antibody levels and cytokine and chemokine concentrations in children with different severe malaria clinical presentations. [file 1475-2875-11-181-S2.pdf]

Additional file 2. Matched comparisons of antibody levels and cytokine and chemokine concentrations in children with different severe malaria clinical presentations.

	**Severe anaemia (N=22 pairs)**		**ARD (N=34 pairs)**		**Prostration (N=50 pairs)**		**Multiple seizures (N=19 pairs)**	
**Parameter**	**Matched comparison**	***P***	**Matched comparison**	***P***	**Matched comparison**	***P***	**Matched comparison**	***P***
**IgG,** *normalized OD*								
MSP-1	-0.1 (-0.4 – 0.3)	0.532	0.1 (-0.5 – 0.3)	1.000	-0.1(-0.5 – 0.3)	0.323	0 (-0.1 – 0.2)	1.000
EBA-175	-0.1 (-0.2 – 0.2)	0.539	-0.03 (-0.3 – 0.5)	0.612	-0.1 (-0.3 – 0.4)	0.194	0.1 (-0.2 – 0.8)	1.000
AMA-1	-0.1 (-0.6 – 0.5)	0.823	0.1 (-0.4 – 0.4)	0.629	-0.1 (-0.4 – 0.3)	0.491	-0.2 (-0.3 – 0.04)	0.175
DBLα	-0.04 (-0.1 – 0.0)	0.031	-0.03 (-0.1 – 0.02)	0.015	-0.03 (-0.1 – 0.02)	0.002	-0.03 (-0.1 – 0.03)	0.256
Pf lysate	0.01 (-0.01 – 0.1)	0.120	0.03 (-0.01 – 0.1)	0.003	0.02 (-0.01 – 0.1)	0.004	0.03 (0 – 0.1)	0.060
**IgM,** *normalized OD*								
MSP-1	-0.2 (-0.5 – 0.1)	0.066	-0.2 (-0.6 – 0.1)	0.217	-0.2 (-0.5 – 0.1)	0.013	-0.2 (-0.4 – 0.1)	0.641
EBA-175	-0.1 (-0.5 – 0.02)	0.055	-0.1 (-0.4 – 0.1)	0.058	-0.1 (-0.4 – 0.2)	0.186	0.03 (-0.3 – 0.2)	1.000
AMA-1	-0.3 (-0.6 - -0.03)	0.004	-0.1 (-0.5 – 0.2)	0.049	-0.1 (-0.4 – 0.1)	0.016	0.03 (-0.5 – 0.2)	1.000
DBLα	-0.1 (-0.3 – 0.2)	0.524	-0.1 (-0.4 – 0.1)	0.228	-0.1 (-0.3 – 0.1)	0.060	-0.1 (-0.4 – 0.3)	1.000
Pf lysate	0.01 (-0.1 – 0.04)	0.815	0.01 (-0.1 – 0.04)	0.230	0 (-0.1 – 0.04)	0.893	0 (-0.1 – 0.04)	0.677
**Cytokines and chemokines,** *pg/mL (TGF- β1 in ng/mL)*								
IL-12p70	-0.2 (-48.6 – 16.4)	0.648	0 (-22.0 – 16.9)	0.693	-0.8 (-25.2 – 12.5)	0.149	0 (-24.3 – 12.5)	0.807
IL-2	0 (-33.9- 13.7)	0.801	0 (-14.6 – 10.8)	1.000	0 (-19.5 – 10.8)	0.613	0 (-14.6 – 10.8)	0.596
IFN-γ	-9.4 (-40.7 – 4.7)	0.134	-4.1 (-20.3 – 10.1)	0.150	-0.1 (-21.0 – 17.2)	1.000	1.0 (-10.4 – 80.3)	0.829
IL-4	-1.2 (-55.6 – 18.5)	0.822	0 (-29.2 – 18.5)	0.644	0 (-25.1 – 17.7)	0.755	0 (-2.4 – 12.2)	0.784
IL-10	-61.2 (-1608.2 – 737.7)	1.000	385.2 (-176.8 – 2492.4)	0.032	151.2 (-672.9 – 1968.4)	0.185	348.5 (-247.9 – 3034.1)	0.073
IL-8	3.8 (-38.0 – 40.4)	0.510	41.4 (-23.1 – 122.0)	0.058	21 (-36.4 – 87.9)	0.174	10.0 (-32.8 – 162.5)	0.633
IL-6	9.7 (-119.1 – 154.4)	0.507	69.5 (-6.3 – 506.6)	0.002	16.1 (-61.0 – 285.7)	0.179	127.1 (-15.0 – 552.9)	0.070
IL-1β	-0.8 (-30.7 – 9.3)	0.834	4.3 (-23.4 – 14.9)	0.214	1.2 (-16.1 – 14.9)	0.581	1.7 (-1.3 – 14.9)	0.152
TNF	0 (-13.8 – 12.7)	1.000	1.9 (-9.6 – 24.8)	0.725	0 (-12.1 – 12.7)	0.509	0 (-8.0 – 0)	0.075
TGF-β1	-106.6 (-237.1 – 19.0)	0.134	-144.2 (-356.4 – 11.9)	0.002	-158.1 (-356.4- 59.2)	0.007	-132.3 (-272.9 – 59.2)	0.166

ARD, acidosis and/or respiratory distress; OD, optical density at 492 nm; *Pf*, *Plasmodium falciparum.*
Data reports the median difference (inter-quartile range) of antibody levels or cytokine and chemokine concentrations between paired children with SM and UM. *P*-values were calculated using Sign test. All comparisons were corrected by the Monte Carlo permutation test (1000 random permutations).
