# Supplementary material for: Low antibodies against Plasmodium falciparum and imbalanced pro-inflammatory cytokines are associated with severe malaria in Mozambican children: a case–control study
Source: Malar J. 2012 May 30;11:181. doi: 10.1186/1475-2875-11-181 (PMC3464173; doi:10.1186/1475-2875-11-181)
Supplement: Additional file 3 — Spearman’s rank correlation coefficients between cytokine and chemokine concentrations in the study population. * P < 0.05. [file 1475-2875-11-181-S3.pdf]

Additional file 3. Spearman's rank correlation coefficients between cytokine and chemokine concentrations in the study population. * *P* <0.05.

	IL-12p70	IFN-γ	IL-2	IL-10	IL-8	IL-6	IL-4	IL-1β	TNF
IFN-γ	0.4378 *								
IL-2	0.4850 *	0.4054 *							
IL-10	-0.0708	0.2249	-0.0733						
IL-8	-0.0922	0.1167	-0.0554	0.4372 *					
IL-6	-0.1546	0.0528	-0.1304	0.8682 *	0.5536 *				
IL-4	0.6926 *	0.5090 *	0.4174 *	-0.0657	-0.0077	-0.1290			
IL-1β	0.4785 *	0.5315 *	0.3390 *	0.3422 *	0.3420 *	0.3247 *	0.6527 *		
TNF	0.3991 *	0.3934 *	0.3021 *	0.3756 *	0.3817 *	0.3516 *	0.4192 *	0.6710 *	
TGF-β1	0.0227	-0.0962	-0.1395	-0.3890 *	-0.2117	-0.3608 *	-0.0714	-0.1834	-0.2656
